# Use of Waste Substrates for the Lipid Production by Yeasts of the Genus *Metschnikowia*—Screening Study

**DOI:** 10.3390/microorganisms9112295

**Published:** 2021-11-04

**Authors:** Andrea Němcová, Martin Szotkowski, Ota Samek, Linda Cagáňová, Matthias Sipiczki, Ivana Márová

**Affiliations:** 1 Faculty of Chemistry, Brno University of Technology, Purkyňova 464/118, 612 00 Brno, Czech Republic; xcszotkowski@fch.vut.cz (M.S.); xccaganova@fch.vut.cz (L.C.); marova@fch.vut.cz (I.M.); 2 Institute of Scientific Instruments of the Czech Academy of Sciences, Královopolská 147, 612 64 Brno, Czech Republic; osamek@isibrno.cz; 3 Department of Genetics and Applied Microbiology, Faculty of Science and Technology, University of Debrecen, Egyetem tér 1, 4032 Debrecen, Hungary; lipovy@gmx.com

**Keywords:** yeasts, *Metschnikowia*, animal fat, lipids, stress factors, Raman spectroscopy

## Abstract

Oleogenic yeasts are characterized by the ability to accumulate increased amounts of lipids under certain conditions. These microbial lipids differ in their fatty acid composition, which allows them to be widely used in the biotechnology industry. The interest of biotechnologists is closely linked to the rising prices of fossil fuels in recent years. Their negative environmental impact is caused by significantly increased demand for biodiesel. The composition of microbial lipids is very similar to vegetable oils, which provides great potential for use in the production of biodiesel. In addition, some oleogenic microorganisms are capable of producing lipids with a high proportion of unsaturated fatty acids. The presented paper’s main aim was to study the production of lipids and lipid substances by yeasts of the genus *Metschnikowia*, to cultivate crude waste animal fat to study its utilization by yeasts, and to apply the idea of circular economy in the biotechnology of *Metschnikowia* yeasts. The work focuses on the influence of various stress factors in the cultivation process, such as reduced temperature or nutritional stress through the use of various waste substrates, together with manipulating the ratio of carbon and nitrogen sources in the medium. Yeast production properties were monitored by several instrumental techniques, including gas chromatography and Raman spectroscopy. The amount of lipids and in particular the fatty acid composition varied depending on the strains studied and the culture conditions used. The ability of yeast to produce significant amounts of unsaturated fatty acids was also demonstrated in the work. The most suitable substrate for lipid production was a medium containing glycerol, where the amount of accumulated lipids in the yeast *M. pulcherrima* 1232 was up to 36%. In our work, the crude animal fat was used for the production of high-value lipids, which to the best of our knowledge is a new result. Moreover, quantitative screening of lipase enzyme activity cultivated on animal fat substrate on selected yeasts of the genus *Metschnikowia* was performed. We found that for the yeast utilizing glycerol, animal fat seems to be an excellent source of carbon. Therefore, the yeast conversion of crude processed animal fat to value-added products is a valuable process for the biotechnology and food industry.

## 1. Introduction

The depletion of fossil fuel energy currently poses an increasing risk to humanity. Therefore, the fuel industry is strongly encouraged to develop alternative energy sources whose composition and use would be more environmentally friendly. Biofuels are one of these renewable energy sources [1]. Various microorganisms such as algae, yeast, bacteria, and fungi are able to accumulate lipids under specific culture conditions. Under certain conditions, microorganisms can produce large amounts of lipids, with their accumulation representing more than 20 to 80% of the total biomass. Various high-value specific chemicals such as Ω-3 fatty acids, fatty alcohols, or carotenes can also be derived from lipid metabolism, which can also be used as food or feed additives [2,3].

Lipids produced by these microorganisms could soon be used as potential storage materials for the future production of specific biofuels. Lipids for these purposes are obtained mainly from oilseeds, animal fats, and waste oils from the food industry. However, these resources are not expected to be able to cover the requirements of the fuel and chemical industries in the future. This is mainly due to concerns about the use of arable land for growing crops for human consumption and food safety [4].

Recently, the use of microbial lipids has been a subject of extensive research aiming to reduce the manufacturing costs associated with fermentation processes. The most important oleogenic microorganisms from the fungal kingdom capable of accumulating more than 20% of their biomass in the form of lipids are ascomycetous, *Basidiomycetous* and *Zygomycetes*. The main representatives of ascomycet oleogenic yeasts include *Yarrowia lipolytica*, *Lipomyces lipofer,* and *Lipomyces starkeyi*, while basidiomycetic yeasts belong mainly to the genera *Phaffia*, *Rhodotorula*/*Rhodosporidium,* and *Sporobolomyces*. Oleogenic yeasts are important producers of lipids, which in the form of oils accumulate inside their cells, while filamentous fungi produce rather longer polyunsaturated fatty acids. The advantage of oleogenic microorganisms lies in their wide variety of substrate uses. In addition to glucose, xylose, glycerol, organic acids, polymeric compounds such as starch, or hydrophobic compounds in the form of alkanes can be used for the production and subsequent accumulation of lipids [2].

Many microorganisms are capable of utilizing free fatty acids regardless of their lipolytic capacity. On the contrary, some microorganisms—mainly yeasts—are able to break down TAGs or their fatty esters. This is due to the active lipase system in their enzymes. It is interesting that some fatty acids that are produced by lipase catalysis are incorporated into the cells at different speeds. Thus, the composition of oils in single cells can be changed by the duration of fermentation [5,6].

The lipid profile of biodiesel varies depending on the type of source material. When terrestrial crops are used for production, the profile is relatively simple. The vast majority are C16-C20 fatty acids, with palmitic acid, stearic acid, oleic acid, linolenic acid, and erucic acid being the majority. Lipids produced by microalgae show greater variability, with the fatty acid content depending on the type of algae used and the culture conditions. Microalgal lipids commonly contain C6-C24 chains. Lipids produced by oleogenic yeasts are composed of C16 and C18 fatty acids. The proportion of palmitic acid (C16) represents approximately 15–25% of the total lipids, while palmitoleic acid (C16: 1) accounts for less than 5% of the lipids by weight. Oleic acid (C18: 1) has the largest proportion in yeast cells, accounting for up to 70% by weight. Stearic acid (C18: 0) is present in relatively small amounts in concentrations of 5–8% by weight and linoleic acid (C18: 2) in concentrations of 15–25% by weight. However, the lipid composition is relative and can vary depending on the type of yeast or even depending on the different strains. The fatty acid profile is also affected by environmental conditions, the growth phase of the microorganism, or the substrate. In general, yeast-produced oils have a similar profile to vegetable oils (e.g., rapeseed and sunflower) [4].

The presented work deals with the screening of various types of culture conditions for lipid production by selected yeast species of the genus *Metschnikowia.* As growth substrate, glycerol and waste fat from animals have been used. This was compared with the optimal glucose medium. Cultivation of glycerol was used for this substrate verification for the production of lipid metabolites. Because animal fat contains glycerol as a major source, we can consider the utilization of this substrate to be important. For yeasts, we followed also the production of lipolytic enzymes, which is related to animal fat utilization and in turn to the production of important lipid metabolites.

Yeasts of the genus *Metschnikowia* occur commonly in nature and in the aquatic environment. Most species are isolated from the terrestrial environment where their immediate occurrence on flowers, fruits, or cuticles of pollinating insects was proved [7,8]. Recently, Raman spectroscopy was used for the initial estimation of lipid production depending on the culture conditions. This method was considered a fast and efficient tool compared to conventional techniques [9].

## 2. Materials and Methods

### 2.1. Strains

Based on our previous study [9], several yeast strains of the genus *Metschnikowia* were selected for cultivation in this study. *Metschnikowia pulcherrima* 29-02-145, *Metschnikowia pulcherrima* 29-02-147, *Metschnikowia pulcherrima* 29-02-149, and *Metschnikowia andauensis* 29-02-129 were obtained from The Culture Collection of Yeast (CCY), Institute of Chemistry, Slovak Academy of Science, Bratislava, Slovakia.

*Metschnikowia chrysoperlae* CBS 9803 (11-1158), *Metschnikowia pulcherrima* CBS 5833 (11-1232), *Metschnikowia fructicola* CBS 8853 (11-1235), *Metschnikowia andauensis* HA 1657 (11-1241), and *Metschnikowia sinensis* CBS 10357 12 were obtained from Centraalbureau voor Schimmelcultures, Delft, (CBS), The Netherlands.

### 2.2. Waste Substrate Processing

Fresh mixed animal fat provided by NorskProtein Co. (Norillia, Norway) was maintained at −20 °C until further use. Fat processing was done according to Reference [10] using mixed samples from different animal sources. The storage time was no longer than 2 weeks. Antioxidant Thermox liquid FG (Food Grade, Des Moines, IA, USA) was added to the fat. The fatty acid composition of the animal fat was verified by NorskProtein. About 80% of the fatty acids were composed from the sum of palmitic acid (23.3%), stearic acid (18.4%), and oleic acid (39.1%), while the summary content of linoleic and linolenic acids represented about 7% [10,11].

The aim of the analysis was to determine the carbon content in the fat, which at a suitable C/N ratio will replace carbon from glucose in the culture media. The total carbon content of the fat derived from triacylglycerols was determined to be 76.78%.

### 2.3. Media and Growth Conditions

Yeast cultures were stored frozen in cryotubes, from which they were transferred to Petri dishes with basic optimal medium. The same optimal medium was used as inoculation medium. It consisted of (g/L) glucose 20; bacteriological peptone 5; yeast extract 10; and salts: KH_2_PO_4_ 1; K_2_HPO_4_ 0.2; NaCl 0.1; CaCl_2_ 0.1; MgSO_4_ 0.5.

Yeasts were cultivated with one-step inoculation followed by production medium. Cultivations were performed in 250 mL Erlenmeyer flasks with a medium volume of 50 mL. The inoculum was cultivated for 24 h with constant shaking at 25 °C. The inoculum was then transferred to the production medium in a volume ratio of 1: 5. The control cultivation was performed under optimal conditions, i.e., in an optimal medium, at 25 °C, with constant shaking for 168 h. Cultivations for lipid production were performed in various production media with constant shaking for 14 days, with the first three days at 25 °C and 11 days at 15 °C.

Lipid-production cultivations differed in the composition of the production media:

1. Cultivational screening of temperature regime: cultivation was performed in 250mL Erlenmeyer flasks in optimal medium with a composition of (g/L) glucose 20; bacteriological peptone 5; yeast extract 10; and salts—KH_2_PO_4_ 1; K_2_HPO_4_ 0.2; NaCl 0.1; CaCl2 0.1; MgSO_4_ 0.5—at laboratory temperature 25 °C (LT) and at reduced (cold) temperature 15 °C (CT).
2. Low-temperature yeast cultivation at different C/N ratios: cultivation was performed in 250mL Erlenmeyer flasks in C/N media ratios of 24, 97, and 150, where glucose or glycerol was used as carbon source and peptone and yeast extract were used as nitrogen source. The medium still contained salts as stated in the optimal medium. For the resulting C/N, the amount of glucose or glycerol in the individual media varied in proportion to the bacteriological peptone and yeast extract. The detailed composition of the carbon and nitrogen source is given in Table 1.

3. Cultivation of yeast at low temperature with the addition of crude fat at different C/N ratios: cultivation took place in 250 mL Erlenmeyer flasks in media with C/N ratios of 24, 97, and 150, where as the carbon source was crude fat (see Section 2.2) with salt added. As a N source, pepton and yeast extract were used, and the C/N value was determined as a ratio of the carbon content in the C source and the nitrogen content in the N source.

The detailed composition of the carbon and nitrogen source is given in Table 2.

All experiments were carried out in triplicate. Cells were collected by centrifugation (5000 rpm, 10 min) for analysis of the biomass and intracellular metabolites.

### 2.4. Analytical Methods

Yeast biomass 10 mL were centrifuged at 5000 rpm g for 10 min to spin down the yeasts. Then, the yeast cells were washed twice with a mixture of distilled water and hexane 1:1 (*v*/*v*) and suspended in 1 mL of distilled water. Then, the purified biomass was quantitatively transferred into Eppendorf tubes, frozen at −20°C, and then freeze-dried. After their weight was determined, to calculate cell dry weight (CDW), the dried cells were used for the analysis of lipids by gas chromatography.

#### 2.4.1. Gas Chromatography Analysis

The total lipids and individual fatty acids were determined by optimized gas chromatography/flame ionization detection (GC/FID) analysis. Lipids were extracted from 10 mg of freeze-dried cells by the Folch method (methanol: chloroform, 2:1) and converted into their fatty acid methyl esters (FAMEs). The FAMEs were analyzed by gas chromatography (GC) analysis. GC analysis of FAMEs was carried out on a TRACETM 1300 Gas Chromatograph (Thermo Fischer Scientific, Waltham, MA, USA) equipped with an Al 1310 autosampler, a flame ionization detector, and a Zebron ZB-FAME column, where the bleed specification at 260 °C was 3 pA (30 m, 0.25 mm, 0.20 µm). The temperature program is introduced in Table 3. Fatty acids were identified by comparison to commercial FAME standards (FAME37, Supelco, Sigma, St. Louis, MO, USA) and quantified by the internal standard method, involving the addition of 100 µg of commercial heptadecanoic acid (Sigma-Aldrich, Merck, Steinheim, Germany). Chromatography data were evaluated by Chromeleon software 7.2 Thermo Fischer Scienfitic, Waltham, MA, USA. The total lipid concentration, based on GC data, was evaluated as well [11].

#### 2.4.2. Determination of Lipase Activity

Enzymatic activity of lipase was determined using a spectrophotometric method based on *p-* nitrophenylpalmitate. Lipolytic enzymes are able to break down this substrate. The product of this reaction is yellow *p-*nitrophenol estimated at 405 nm using spectrophotometry. For the production of the calibration curve, the concentration 0.5 mmol/L of *p-*nitrophenol was used. The calibration set for p-nitrophenol—ranging from 0.005 to 0.05 mmol/L—was prepared by diluting stock solution (50mM Tris-HCl) with a buffer of pH 8.5.

#### 2.4.3. Raman Analysis and Data Processing

To prepare samples for Raman analysis, the following steps were addressed. One milliliter of yeast culture was transferred from the cooling box into a 1.5 mL tube, and the cells were centrifuged (10,000 rpm, 2 min). In the next step, approx. 20 µL of cell pellets formed by centrifugation was pipetted onto a CaF (Raman grade) microscope slide. The cell suspensions were analyzed using Raman micro spectrometer.

The yeast suspension was analyzed using the Renishaw inVia system (Renishaw inVia Raman Spectrometer, Renishaw plc., Wotton-under-Edge, UK), with 785 nm single-mode diode laser as the excitation source. The laser beam was focused onto a sample by the microscope objective (Leica, Wetzlar, Germany, 50×, NA (Numerical aperture) 0.5) with the laser spot diameter of approximately 2 μm × 10 μm (note that such laser spot shape is characteristic for the Renishaw inVia instrument), with a full axial depth of the excitation region at 8 µm. The laser was focused onto the surface of the sample. Overview spectra were acquired in the range of 700–1800 cm^−1^. Each spectrum used for calibration was measured for 15 s.

The Raman spectra were treated with the Savitzky–Golay procedure coupled with an advanced rolling filter background removal routine (using the program written in-house using MatLab software (MathWorks, Natick, MA, USA)) [12,13].

### 2.5. Statistical Analysis

The growth experiments in Erlenmeyer flasks were carried out in triplicate. The presented results are the mean of the replicates from cultivation and GC analysis, and the standard deviations are shown in the tables. Statistics were performed using the Excel software package (Microsoft Excel 2013, Microsoft Corp., Redmond, WA, USA).

## 3. Results and Discussion

Oleogenic yeasts are organisms defined by the ability to accumulate lipids, which represent a significant portion (20–70%) of their total cell mass. Yeast lipid production is usually conditioned by limitation to one of the essential nutrients, while the presence of a carbon source is in excess. Nitrogen limitation is considered to be the most common factor that triggers microbial lipid synthesis, but sulfur, phosphorus, or zinc may also be this limiting factor [14,15].

However, it has only been shown in recent years based on experimental studies that by manipulating the culture conditions and also by using different culture substrates, increased lipid production can be achieved in the yeast *Metschnikowia pulcherrima*, and this yeast has been defined as oleogenic [2,16,17,18]. In a previous study [9], we found that low temperature has a direct effect on increased lipid production. In this screening study, we focus on the lipid profile of yeast fatty acids cultured in two temperature regimes, laboratory temperature (LT −25 °C) and cold temperature (CT −15 °C). We verified the utilization of glycerol by yeasts and their lipid production. In our work, the crude mixture of animal fat was used for the production of high-value lipids, which is to the best of our knowledge a new result. Thus, for yeast utilization, glycerol animal fat seems to be an excellent source of carbon.

### 3.1. Screening of Temperature Regime for Lipid Production in Metschnikowia Strains

Based on an experimental study, it was found that one of the factors that significantly affect not only biomass yield but also lipid production is temperature. Increased lipid accumulation has been shown to be positively affected by lower temperatures compared to room temperature. This fact also seems to be advantageous in terms of sterility, as lower temperatures are not optimal for the growth of most microorganisms, especially bacteria [2].

Yeasts cultivated at different temperatures than the optimum one are exposed to temperature stress. Temperature stress causes various changes in yeast metabolism, which affects their growth and production properties [9]. The influence of temperature stress due to low temperature (CT −15 °C) compared to the laboratory (LT −25 °C) on *Metschnikowia* yeast was monitored on optimal glucose media with decreased C/N ratio (you can see at Section 2.3).

Cultivation of yeasts that had been kept at laboratory temperature for the whole time resulted in higher biomass production, compared to cultivation in environments with low temperature (Table 3). The low values of the produced biomass were generally lower in the *Metschnikowia* yeast, but on the other hand, the change in temperature did not significantly affect them. Slowed metabolism and division of yeast cells by reduced temperature resulted in increased lipid production in all studied *Metschnikowia* strains. Yeasts (Table 3) show the same trend as in the previous work [9].

#### 3.1.1. Lipid Overproduction in *Metschnikowia* Strains at Low Temperature

Changing the temperature regime of the cultivation towards lower temperatures had a positive effect on lipid production, as higher yields of lipids were achieved in all yeasts cultivated at low temperatures. As shown in Table 4, higher biomass production was most often achieved by cultivation at room temperature. Overall, most lipids were contained in the cells of the yeast *M. andauensis* 1241 (7.9%) at reduced temperature, which also made it the highest producer of lipids compared to other yeasts.

The fatty acid profile was determined on laboratory (LT) and reduced temperature (CT) control media where glucose was the main carbon source.

**Figure 1:**
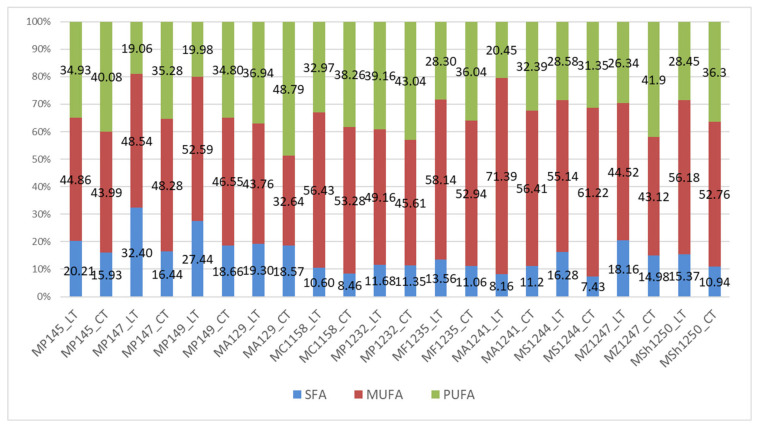
The percentage of saturated (SFA), monounsaturated (MUFA), and polyunsaturated (PUFA) FA in selected yeast strains grown at laboratory temperature (LT) and low cold temperature (CT). Legends: LT means 25 °C, CT means 15 °C, MP145—*Metschnikowia pulcherrima* 145, MP147—*Metschnikowia pulcherrima* 147, MP149—*Metschnikowia pulcherrima* 149, MA129—*Metschnikowia andauensis* 129, MC1158 – *Metschnikowia chrysoperlae* 1158, MP1232—*Metschnikowia pulcherrima* 1232, MF1235—*Metschnikowia fructicola* 1235, MA1241—*Metschnikowia andauensis* 1241, MS1244—*Metschnikowia sinensis* 1244*,* MZ1247—*Metschnikowia zizyphicola* 1247, MSh1250—*Metschnikowia shanxiensis* 1250.

The influence of low temperature on the composition of fatty acids in microbial lipids was evident in all the yeasts studied. Results described in Figure 1 show a stable trend of increased production of SFA in laboratory temperature between the majority of studied *Metschnikowia* strains. This phenomenon is most markedly observed in the strains *Metschnikowia p.* 147, *Metschnikowia s.* 1244, and *Metschnikowia p.* 149, where the highest production of 32.40% and 27.44% was achieved. In the case of other studied strains, the difference in SFA production depending on the cultivation temperature was lower and ranged from 0.5 to 4.0%. The application of reduced temperature had a positive effect on the increased accumulation of unsaturated fatty acids on the expense of SFA and MUFA. The results show that the increase in UFA in most strains is primarily in favor of the formation of polyunsaturated fatty acids. The highest PUFA content, more than 40%, was measured in *Metschnikowia p.* 145, *Metschnikowia a.* 129, *Metschnikowia p.* 1232, and *Metschnikowia p.* 1247.

Oleic acid had the highest concentration in the yeast cells. In some strains, it accounted for more than half of the fatty acid profile. However, a decrease in oleic acid content of almost 17% was observed in the cultivation at reduced temperature (CT). On the other hand, the amount of linoleic acid increased. Thus, it can be concluded that lower temperature conditions are likely to be more suitable to produce polyunsaturated fatty acids, mainly linoleic acid. A-linolenic acid was also formed in a small proportion. The low temperature has been shown to be a supporting factor for the increase in polyunsaturated fatty acids in almost all the cultivations studied.

Table 4 shows the fatty acid profile of *M. andauensis* 1241, an dthe majority of the lipids produced in both temperature regimes had oleic acid. However, when using a low temperature, we can observe a significant decrease in oleic acid and increase in linoleic acid as compared to the culture at room temperature.

The low temperature even enhanced the production of other significant unsaturated FA that were not produced at room temperature at all, such as γ-linolenic acid, eicosadienic acid, arachidonic acid, or eiosapentaenoic acid in all the yeasts studied.

The effect of temperature on lipid production demonstrated in this work is consistent with the results of the study, where the stimulating effect of reduced temperature on increased lipid production was also demonstrated [2,9].

In addition, it is necessary to highlight the very significant effect of reduced temperature on the composition of FA of microbial lipids. These changes in the fatty acid composition are probably closely related to the effect of low temperature on the membranes, which reduces their fluidity.

The effect of the temperature regime on lipid profile was therefore screened in addition to the optimal glucose medium with low C/N ratio also in media with increased C/N ratio, namely 97 and 150 in selected yeast strains.

**Figure 2:**
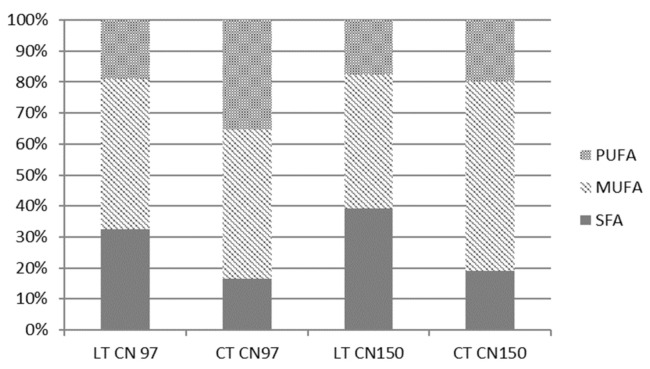
Influence of temperature regime and C/N ratio 97 and 150 on fatty acid production (*M. pulcherrima* 147).

For better clarity for the reader, the results are not shown except for in Figure 2. In general, however, low temperature (CT) and an increased C/N ratio caused increased production of MUFA and PUFA in *Metschnikowia* yeasts. Figure 2 demonstrates the changes in saturation and unsaturation of fatty acids (FA) in the yeast *Metschnikowia pulcherrima* 147. Both in the previous section (3.1.1) and this section, the cold temperature had an effect on the increase in PUFAs, in particular linoleic acid, compared to laboratory temperature, in cultures at all C/N ratios. Additionally, the cold temperature had the effect of reducing SFAs compared to laboratory temperature at all C/N ratios.

The effects of temperature and C/N ratio on the production properties of the studied *Metschnikowia* yeast proved to be significant and was demonstrated in all studied yeast cultures.

The results obtained show that with increasing C/N ratio, the amount of MUFA also increases at cold temperature. If we want to biotechnologically focus on higher PUFA production in yeasts of the genus *Metschnikowia*, we should choose a cold culture temperature and a C/N ratio of 97 or close to 100.

#### 3.1.2. Combined Effect of Low Temperature and C/N ratio on Lipid Production

Lipid production in oleaginous yeast is influenced by several factors such as carbon and nitrogen source, C/N ratio, medium aeration, temperature, and pH. Different studies have therefore sought to optimize culture conditions to achieve the highest possible lipid production in yeast cells [2]. The biomass production and increased lipid accumulation in oleaginous yeasts are opposing phenomena that are controlled by a number of external and internal cultivation factors such as aeration, sufficient nutrients, and temperature.

Based on the results of increased lipid accumulation at a reduced temperature [9], the experiment continued by examining the effect of the C/N ratio on lipid production. Lipid accumulation is usually optimal at a C/N ratio higher than 65 and close to 100 [2]. The results of combined low temperature and different C/N ratios on lipid accumulation in multiple yeast species are shown in Table 5.

The production of biomass (g/L) and percent content of fatty acids per biomass weight (%) has been monitored in each cultivation for the chosen yeast strains according to the used type of carbon source (glucose) and chosen C/N ratio.

*Metschnikowia* yeast strains were cultivated in media where glucose was used as the main carbon source. Different contents of peptone and yeast extract were used in each medium as a source of nitrogen depending on the selected C/N ratios of 24, 97, and 150.

The results clearly show that as the C/N ratio increases, the amount of biomass in almost every yeast strain decreases. However, these differences are not so significant for most strains and range from 0.2–1.0 g/L. A significant decrease in biomass production with increasing C/N ratio was observed in four strains of *M. chrysoperlae* 1158, *M. pulcherrima* 1232, *M. fructicola* 1235, and *M. zizyphicola* 1247. In the case of these strains, the decrease in biomass production at the highest C/N ratio was more than 2 g/L compared to the lowest C/N ratio. Lipid production increases with increasing C/N ratio in all strains. By cultivating the yeast on glucose media with a C/N 97 ratio under reduced temperature conditions (15 °C), a relatively high lipid production was achieved. In almost half of the strains used, lipid production was more than 12% by volume of biomass.

By increasing the C/N 150 ratio in the glucose medium, relatively high microbial lipid productions were also observed. Some strains achieved almost 20% lipids from the dry weight of biomass; in particular, the yeast *M. andauensis* 1241 reached 19.6 ± 1.6% lipids in the production of 8.2 g/L biomass. This phenomenon was caused by the lower content of nitrogen source in the C/N 150 medium, which led to its rapid depletion, and the cells stopped their growth and began to accumulate lipids.

There were no significant variations in lipid or biomass production on both culture media. From this we can conclude that the yeast showed a similar trend on media with the same substrate. When the C/N ratio was changed, only the percentual lipid content and biomass production changed. In general, at higher C/N ratios, yeast showed a higher percentage of microbial lipid production [9].

### 3.2. Screening of Lipid Production in Stressed Metschnikowia Strains Grown on Glycerol as a Carbon Source

More than 95% of the raw materials for biodiesel production come from edible oils, which is the cause of deforestation in some countries, due to the increase in required agricultural land. The main by-product of biodiesel is crude glycerol, but refining crude glycerol to pure glycerol is very expensive. According to research from 2016, crude glycerol can be used as the main carbon source for lipid-producing organisms [17,19,20].

In the next part of the work, therefore, the carbon source of glucose was replaced by glycerol. The other conditions were maintained as in the previous part of the study, namely low temperature and C/N ratios of 24, 97, and 150.

Relatively high biomass production in g/L was recorded for all strains cultivated on glycerol medium with C/N 24. The most biomass with a volume of 27.7 g/L was produced by the yeast *M. sinensis* 1244 (Table 6), which was more than 2 times higher than glucose media. The results show that in general, glycerol is a better carbon substrate in terms of biomass production.

However, despite the same amounts of biomass, these strains did not show identical production properties in terms of lipid formation. In the strain *M. zizyphicola* 1247, up to 35.5% of lipids from the total biomass content were synthesized, which was also the highest measured value of lipid content from the total biomass.

In the monitoring of lipid production by the yeasts, *M. pulcherrima* 145*,* 147*,* and *M.andauensis* 129 on glycerol media, there were no significant deviations depending on the ratios of C/N used, namely 24, 97, and 150. Compared to the previous cultivation of yeast on glucose as a carbon source, there were no significant changes in the production of either biomass or lipids, as is the case with other monitored yeast strains.

In these other strains, in comparison with media where glucose was the main carbon source, higher biomass yields were achieved on glycerol and at the same time higher yields of microbial lipids. Thus, it can be argued that the glycerol content effectively promoted microbial growth in most of the strains studied.

The use of glycerol as a waste substrate therefore has the great advantage of its easy availability and low cost. At the same time, its use shows better production properties.

In the biotechnological process, glycerol serves as one of many potential sources to produce microbial lipids by oleogenic microorganisms. Therefore, the ability to utilize pure or crude glycerol has been the subject of several studies. The yeast *M. pulcherrima*, whose lipid content was determined to be almost 40% after 14 days of cultivation under reduced conditions (15 °C), was also subjected to such studies. This confirmed that the yeast is able to grow and at the same time utilize the glycerol contained in the media in high concentrations [2].

In the present paper, the best production properties were achieved by the yeast *M. pulcherrima* 1232, while the content of accumulated lipids in its cells was up to 36.31%. Very good yields of lipids from biomass also showed *Metschnikowia sinensis* 1244 for all measured media. However, maximum lipid production of nearly 30% was achieved for media with a C/N ratio of 150. For *M. zizyphicola* 1247, the maximum was at 35.5% for media with a C/N ratio of 24.

Compared to the cited study, no significant difference in the amount of lipids produced is observed. The mutual comparison of studies and the results of the work proves that the C/N ratio, temperature conditions, and the cultivation time itself significantly affect the production of lipids in oleogenic yeasts. High lipid yields on media where glycerol acted as the primary carbon source have been demonstrated in other yeasts, such as e.g., *Yarrowia lipolytica*, *Candida curvata,* or *Rhodotorula graminis*. By cultivation of glycerol, microbial lipid yields represented between 28 and 53% of the biomass content [17]. Glycerol is an important carbon source for the yeast of the genus *Metchnikowia* targeted for the biotechnological production of high-value lipids. Thus, we can consider that with targeted conversion of lipid-rich waste—such as animal fat, which is a rich source for TAG—we can contribute to the reduction of the burden on the environment by decreasing the amount of waste substrates.

#### Monitoring of Fatty Acid Profile in Stressed Metschnikowia Strains

The fatty acid profile of the individual strains cultivated on glucose and glycerol as the main carbon source was determined by gas chromatography. Depending on the type of medium used and the C/N ratio, the production strains of the examined strains were compared with each other.

The results of the analysis of the fatty acid produced by *Metschnikowia* yeasts cultivated on different C/N ratios show a stable production of individual types of fatty acids in all studied yeast strains on both types of carbon sources. As the amount of carbon in the medium increases, we observe a slight increase in the content of unsaturated fatty acids. Thus, it can be stated that increasing the amount of carbon in the medium has a positive effect on the production of unsaturated fatty acids, but only to a certain extent. On media with a large excess of carbon (C/N 150), the results show the opposite effect, where the production of unsaturated fatty acids decreases to values comparable to media with a low C/N ratio or even less. The complete GC results describing the content of fatty acids are shown in Tables S1 and S2 in the Supplementary Material.

The majority of fatty acids produced by *Metschnikowia* yeasts cultivated on both glucose (Section 3.1.2) and glycerol (Section 3.2) media with the same C/N ratio was oleic acid and linoleic acid. The use of glycerol as a carbon source led to higher yields of linoleic acid compared to glucose medium. Palmitic acid, palmitoleic acid, and stearic acid accounted for a significant proportion of fatty acids. In addition, it can be seen immediately that the yeast profile cultivated on glycerol is more diverse, enriched with significant unsaturated FA such as α-linolenic acid or arachidonic acid.

**Figure 3:**
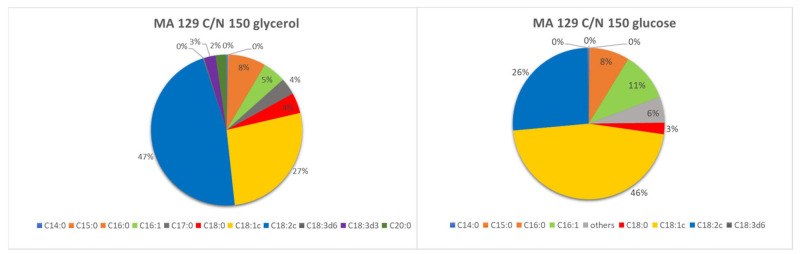
Fatty acid profile of *M. andauensis* 129 (MA 129) cultivated at C/N ratio 150 on various carbon sources at cold temperature.

The yeast *Metschnikowia andauensis* 129 (MA129) differed in the proportion of fatty acids at the same C/N ratio but different carbon sources (Figure 3). While oleic acid, linoleic acid, and palmitoleic acid predominated in the glucose medium, linoleic acid predominated in the glycerol medium. Complete results of fatty acid profile analysis are listed in Tables S1 and S2.

Cultivation on glycerol medium resulted in gamma and alpha-linolenic acid, which did not occur during cultivation on glucose. Although the fatty acid profile of the yeast *M. andauensis* 129 was strongly dependent on the carbon substrate used, the individual C/N ratios studied (24, 97, and 150) did not significantly affect it, and thus the proportions on the individual glycerol and glucose media are very similar.

The other studied strains of *M. pulcherrima* (145, 147, and 149) showed a higher dependence of the FA profile on the ratio of carbon and nitrogen in the medium compared to *M. andauensis* 129. The yeasts of genus *Metschnikowia* showed a C/N ratio of 97 on glycerol as the most preferred for linoleic acid formation, as well as to glucose media.

**Figure 4:**
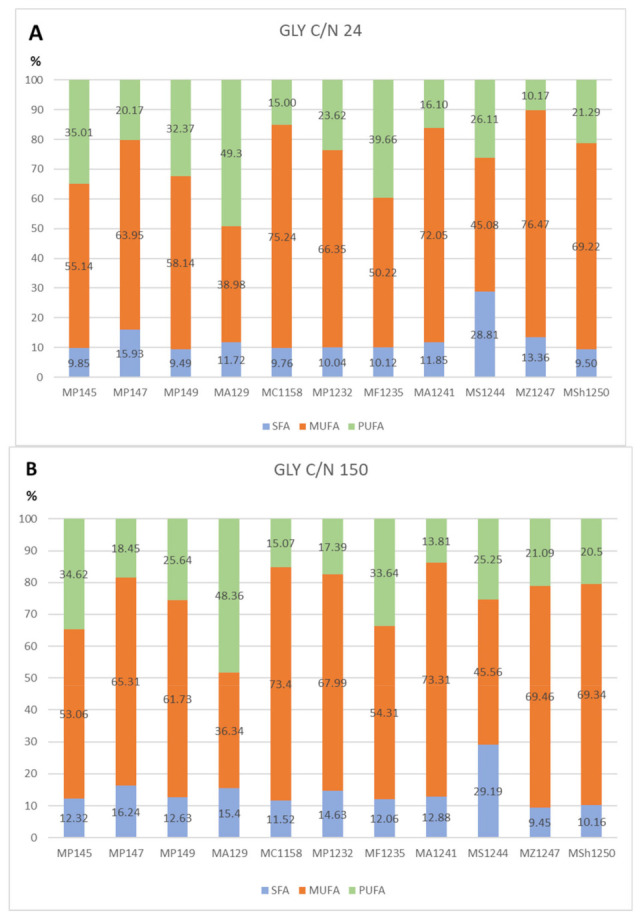
Percentage of fatty acids of yeasts on glycerol medium with C/N ratios 24 (**A**) and 150 (**B**). Legends: *Metschnikowia pulcherrima* 145 (MP145), *Metschnikowia pulcherrima* 147 (MP147), *Metschnikowia pulcherrima* 149 (MP149), *Metschnikowia andauensis* 129 (MA129), *Metschnikowia chrysoperlae* 1158 (MC 1158), *Metschnikowia pulcherrima* 1232 (MP 1232), *Metschnikowia fructicola* 1235 (MF 1235), *Metschnikowia andauensis* 1241 (MA 1241), *Metschnikowia sinensis* 1244 (MSi 1244), *Metschnikowia zizyphicola* 1247 (MZ 1247), *Metschnikowia shanxiensis* 1250 (MSh 1250).

**Figure 5:**
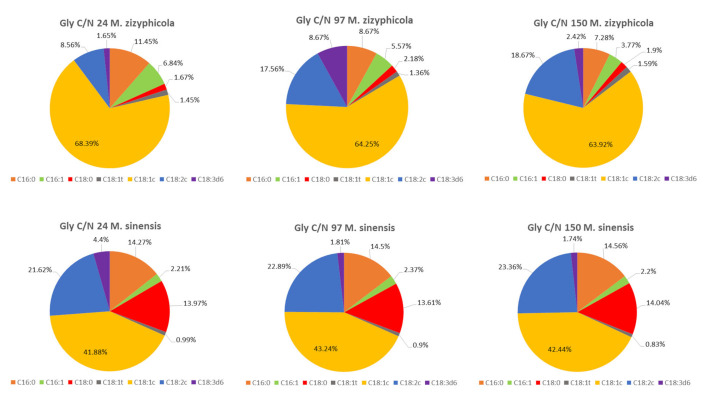
Fatty acid profile of *M. zizyphicola* 1247 and *M. sinensis* 1244 on glycerol media with different C/N ratios (24, 97 and 150). C16:0-palmitic acid; C16: 1-palmitoleic acid; C18:0 - stearic acid; C18: 1t-elaidic acid; C18: 1c-oleic acid; C18: 2c-linoleic acid; C18: 3d3-α-linolenic acid.

All strains showed a very similar fatty acid profile when growing in glycerol media with various C/N ratios (Figure 4A,B see selected C/N ratio 24 and 150). The only exception was the yeast *M. sinensis* 1244 (Figure 4 and Figure 5), for which the production of some types of fatty acids was different. As the only strain, it was able to produce the highest content of palmitic and stearic acid on all glycerol as well as glucose media. At the same time, it was also the one of lowest producers of oleic acid in these media. Thus, the difference in fatty acid production could probably be related to the genetic makeup of this yeast. The most common acids were oleic acid and linoleic acid. Palmitic, stearic, and palmitoleic acids also formed a certain proportion of the profile. Alpha-linolenic acid was also present in glycerol media, which was not in the fatty acid profile of glucose media.

The results of other cultured strains of *Metschnikowia* yeast show a stable trend of fatty acid production. Even in media with different C/N ratios, the yeast maintains a relatively stable FA profile, which varies only in the range of 1–5%. In general, with an increased C/N ratio, we observe a slight increase in PUFA production at the expense of MUFA (Figure 4 and Figure 5).

As shown in Figure 5 the production of oleic acid by increasing C/N in the yeast *M. zizyphicola* 1232 tends to decrease. As C/N increased, so did the content of palmitic and palmitoleic acid. However, the positive effect of increasing C/N was demonstrated in the formation of linoleic acid, where the % proportion of acid also increased with increasing C/N. The direct effect of C/N on stearic acid production has not been demonstrated, as there has been no decrease or increase in C/N production with increasing C/N. More results are shown in Table S2.

An increase or decrease in the C/N ratio did not have a direct effect on oleic acid production in the yeast *M. sinensis* 1244. However, the increasing C/N ratio had a favorable effect on the production of palmitic and linoleic acid, the content of which increased proportionally even with increasing C/N. Stearic acid did not differ in amount in different media but reached the highest values in all studied yeasts of the genus *Metschnikowia* when cultivated on glucose or glycerol at different C/N ratios. Compared to glucose media, with the biotechnological focus on increased PUFA production in *Metschnikowia* yeasts, there is no marked difference between C/N ratios of 97 and 150 on glycerol media. Thus, it can be concluded that the yeasts of the genus *Metschnikowia* are very stable in the area of fatty acid production and are not affected by the different C/N ratios. As a result, mainly the total lipid production and the associated fatty acid production can be increased by increasing the C/N ratio. By exchanging a simple carbon source, the fatty acid profile can then be modulated to some extent.

### 3.3. Production of Lipids in Metschnikowia Strains Cultivated on Crude Animal Fat as a Carbon Source

With the growing expansion of the meat industry, more and more animal fat is being produced as a by-product, which is currently considered to be one of the main sources in biodiesel production [21,22,23]. From a chemical point of view, animal fats are triacylglycerols in which glycerol is esterified with three fatty acids. Animal fats are characterized by a higher content of saturated fatty acids or long chain fatty acids [24].

Again, the media were prepared to maintain the selected C/N ratio, and in particular 24, 97, and 150 ratios at cold temperature were used (see Section 2.3)

In the comparison to glucose and glycerol media, the relatively lowest biomass yields were achieved on fat media at the C/N ratio 24–150 (Table 7), similar to the study [11] where crude fat was used to cultivate carotenogenic yeast. Limitation of the nitrogen source in C/N 150 fat media slightly increased lipid accumulation in yeast cells. In the fat medium C/N 97, the yeast *M. andauensis* 1241 showed the highest lipid production. The lipid content was 14.4% by weight of the biomass.

The results confirm that all studied strains of *Metschnikowia* yeast were able to utilize crude animal fat. All yeasts were able to accumulate more than 5% lipids in their cells. The yeast *M. pulcherrima* 1232, which achieved very good production on glycerol media, was rather average on fat media. Additionally, the yeast *M. sinensis* 1244, a good producer of glycerol media, achieved the lowest production of lipids on fat media with C/N ratio 150.

In the comparison of fat media, the relatively lowest biomass yields were achieved at a C/N ratio of 150. Limitation of the nitrogen source in C/N 150 fat media slightly increased the lipid accumulation in yeast cells. Overall, of all fat media at the C/N ratio of 150, the largest amount of lipids was recorded, around 20.35% in the yeast *M. zizyphicola* 1247.

The lowest biomass production was recorded by cultivation on waste fat substrate. This phenomenon may have been since the yeast was probably not able to utilize pure fat in sufficient quantities due to its solid state in cultivation conditions. The carbon contained in the substrate was difficult for them to access, which was reflected in the amount of biomass produced. In order to make the fat more accessible to yeast, it would be appropriate to use an emulsifier in the future or to modify the fat technologically, for example by hydrolysis. After hydrolysis of the fat, carbon is released into the medium mainly in the form of fatty acids. A minor proportion is glycerol, which can serve as a rapid source of energy for yeast sufficient for the initial growth of biomass. Subsequently, yeast metabolism focuses on fatty acid processing.

The fatty acid profile accumulated in the cells was monitored in all studied yeasts of the genus *Metschnikowia* using the gas chromatography technique. The fatty acid profile was compared between strains according to the C/N ratio of the medium used.

The presence of palmitic and palmitoleic acid was confirmed in all strains examined by cultivation on waste fat with a C/N ratio of 24. Stearic acid was also present in small amounts in all yeasts. Of all the fatty acids, oleic acid was the most produced. In the cells of some yeasts, its content was up to 74.82% (*M. zizyphicola* 1247), 74.77% (*M. chrysoperlae* 1158), and 72.92% (*M. pulcherrima* 145) of total fatty acids. When cultivated on C/N 24, the oleic acid content of all strains always accounted for more than half of the fatty acid content. As in glycerol cultures, yeasts of the genus Metschnikowia have shown the ability to produce linoleic and α-linolenic acid on the fat medium. The content of α-linolenic acid ranged from 0.83% (*M. pulcherrima* 147) to 4.68% (*M. sinensis* 1244).

As with the lower C/N medium, yeast when cultivated at a C/N ratio of 97 showed a similar trend in fatty acid production. Again, the presence of oleic, palmitic, stearic, and palmitoleic acids was detected in all yeasts examined. As with C/N 24 and C/N 97, an increased content of Ω-3 fatty α-linolenic acid was observed in *M. sinensis* strain 1244 among other yeasts. At C/N 24, the content of α-linolenic acid was 4.68%, and at C/N 97, the content slightly increased to 5.93%.

**Figure 6:**
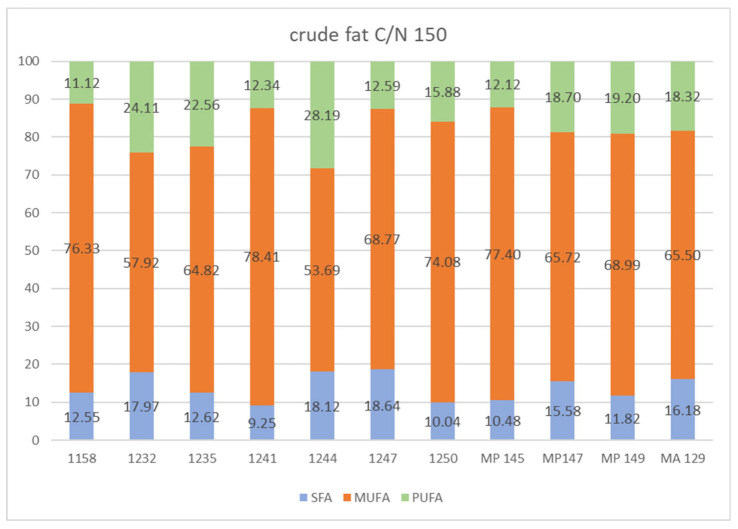
The percentage of saturated (SFA), monounsaturated (MUFA), and polyunsaturated (PUFA) fatty acids (FA) Legends: *Metschnikowia chrysoperlae* 1158 (MC 1158), *Metschnikowia pulcherrima* 1232 (MP 1232), *Metschnikowia fructicola* 1235 (MF 1235), *Metschnikowia andauensis* 1241 (MA 1241), *Metschnikowia sinensis* 1244 (MSi 1244), *Metschnikowia zizyphicola* 1247 (MZ 1247), *Metschnikowia shanxiensis* 1250 (MSh 1250), *Metschnikowia pulcherrima* 145 (MP145), *Metschnikowia pulcherrima* 147 (MP147), *Metschnikowia pulcherrima* 149 (MP149), *Metschnikowia andauensis* 129 (MA129).

Figure 6 shows the content of fatty acids present in the biomass of yeast cultivated on a fat medium in C/N 150. The production of palmitic, palmitoleic, and stearic acids was recorded on this medium. Relatively high palmitic acid production was observed in *M. pulcherrima* 1232 *strain* compared to the yields obtained on the medium with C/N 97. The highest yield of palmitic acid was obtained on the fat medium with C/N 150. Relatively increased yields were also observed for stearic acid production ranging from 0.88% (*M. andauensis* 1241) to 14.06% (*M. zizyphicola* 1247). Oleic acid had the highest proportion of all fatty acids. The oleic acid content of *M. pulcherrima* 145 was up to 71.42%. It can also be seen from Figure 6 that the presence of α-linoleic acid was detected in a smaller amount in the cells of all examined yeasts, but it was mostly contained again in the yeast *M. sinensis* 1244 (5.67%).

From the results given above and in Figure 6, which summarize the proportion of saturated and unsaturated fatty acids in the obtained microbial lipids, it can be clearly stated that in all strains of yeasts of the genus *Metschnikowia* there was a significant predominance of monounsaturated fatty acids.

The obtained results demonstrate the ability of all monitored strains of yeasts of the genus *Metschnikowia* to use waste animal fat for their growth and lipid production.

**Figure 7:**
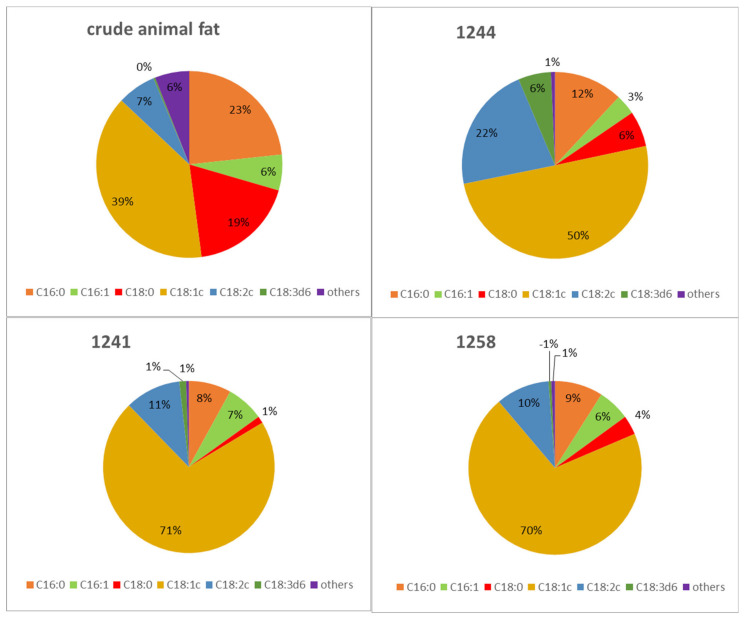
Conversion of crude animal fat to others lipid metabolites by selected *Metschnikowia* strains (cultivated on crude fat medium with a C/N of ratio 150) Legends: (1244) *Metschnikowia sinensis* 1244*,* (1241) *Metschnikowia andauensis* 1241*,* (1158), *Metschnikowia chrysoperlae* 1158. C16:0-palmitic acid; C16: 1-palmitoleic acid; C18:0 - stearic acid; C18: 1-oleic acid; C18: 2-linoleic acid; C18: 3-α-linolenic acid.

As mentioned above, the fatty acids from crude animal fat were composed of the sum of palmitic acid (23.3%), stearic acid (18.4%), and oleic acid (39.1%), while the summary content of linoleic and linolenic acids represented about 7% [11]. Figure 7 shows conversion of animal fat into other lipid metabolites for selected yeasts of the genus *Metschnikowia*. The content of selected fatty acids is different from the original animal fat and is dependent on the given strain.

Using different biotechnological applications, the potential to grow on raw animal fat for the selected yeasts of the genus *Metschnikowia* was investigated. For this carbon source, several strains showed specific growth, biomass, and lipid production, which was higher compared to glucose or glycerol medium. Moreover, monitoring of the fatty acid profile suggests that animal fat could be used as a source of carbon in yeast cultivation for biomass production and conversion to other types of fatty acids. More data and dedicated results are mentioned in Table S3 in the Supplementary Material.

As can be seen from Table S3, the conversion of animal fat by yeast increased the amount of oleic and linolenic acid; oleic acid in turn became the most abundant fatty acid. Compared to the original crude animal fat, which contained a relatively high amount of saturated fatty acids, namely stearic and palmitic, for selected yeasts, the content of these acids is lower. The only strain that differs from other yeast strains is again *Metschnikowia sinensis* 1244, which produces higher amounts of stearic acid compared to the other strains. However, this strain has the highest amount of polyunsaturated fatty acids. We would like to note that while crude animal fat contains approximately 20% stearic acid, this fatty acid is significantly degraded in yeast metabolism and converted to other lipid metabolites. The highest potential in the transformation of waste fat with a high SFA content compared to MUFA was observed in strains *M. chrysoperlae* 1158, *M. andauensis* 1241, and *M. pulcherrima* 145, where these strains accumulated more than 70% oleic acid. In contrast, in *M. pulcherrima* 1232, *M. fructicola* 1235, and *M. sinensis* 1244 strains, the transformation of SFA to unsaturated fatty acids also focuses on the formation of PUFA, which reaches more than 20% (C/N 150). The results show that the ability of yeast to transform saturated fatty acids from waste fat to unsaturated fatty acids increases with increasing C/N ratio. Thus, utilization of animal fat by yeasts of the genus *Metschnikowia* seems to be very promising for different biotechnological applications. Additionally, the production of various lipid metabolites depending on the strain and culture conditions is possible.

If we compare the results of *Metschnikowia* strains grown on glycerol or waste fat with other yeast species, we see that lipid production is relatively lower. The genus *Yarrowia* is able to produce 45–55% lipids on waste lipid-based media [25,26]. Representatives of carotenogenic yeasts, e.g., the genus *Sporidiobolus* [11], are also able to achieve identical productions of up to 55% on lipid wastes.

However, unlike the above-mentioned species *Yarrowia* or *Sporidiobolus*, the main biotechnological significance of the genus *Metschnikowia* stems from its genetic makeup and ability to transform and synthesize higher fatty acids. These yeasts are thus able to transform wastes with a high SFA content into microbial oils with a high content of PUFA and MUFA. In the case of red yeast, a suitable waste substrate such as coffee oil must be used to achieve biomass with a wide range of unsaturated fatty acids [11].

### 3.4. Production of Lipolytic Enzymes by Yeast of the Genus Metschnikowia Cultivated on Animal Fat Substrate

Yeast biomass production in general on waste fat was relatively low. The reason for the low production of fatty media could probably be the low concentration of waste fat, which did not represent a sufficient amount of carbon in the medium, or the carbon contained in the substrate was difficult to obtain for them. However, it should be noted that yeasts of the genus *Metschnikowia* were in the past included in the genus *Candida*, based on several similarities between these currently two genera [27]. Some yeasts of the genus *Candida* are known to overproduce lipases [28]. In the present study, it was hypothesized that yeasts of the genus *Metschnikowia* could also show increased lipase activity, which could be related to the ability to easily degrade the animal fat present in the medium, utilize it, and accumulate lipids at the same time. By using waste fat with yeast, we can obtain new biotechnologically important products from processed industrial waste and at the same time reduce the burden on the environment [29,30].

It was proved that the yeasts of the genus *Metschnikowia* showed a potential ability to utilize waste substrates in the form of animal fat. Thus, in the next step, we monitored their ability to produce hydrolytic enzymes, namely lipases. Quantitative screening of lipase enzyme activity was performed on selected yeasts of the genus *Metschnikowia* using a p-nitrophenyl palmitate spectrophotometric method [31]. Consequently, the amount of p-nitrophenol released by the action of the enzyme on p-NPP was measured. The lipase activity of the individual strains was expressed in units of nmol/mL·min. Culture media containing waste fat as the main carbon source were used to determine the enzymatic activity of *Metschnikowia* yeast lipases. The culture media were prepared as the optimal medium (see Section 2.3), where instead of glucose a reference amount of fat (47 g/L) was added as a carbon source. In addition, an emulsifier in the form of polysorbate 80 (Tween 80) was added to one culture series containing fat. In order to increase the activity of extracellular lipases, surfactants are often added to the medium [32,33]. In the present work, Tween 80 (a non-ionic polyoxyethylene detergent) was used to investigate lipase activities, which stimulates the biosynthesis and secretion of lipases because it increases cell permeability and enables lipase export across the cell membrane. Table 8 shows a comparison of the lipolytic activity which selected yeast samples showed on fat media and on media where Tween 80 was added. Lipase production was confirmed in all strains under investigation.

It can be seen from Table 8 that overall, the lowest enzymatic activity of lipases on the unmodified waste fat was recorded for *M. zizyphicola* strain 1247 (0.57 nmol/min mL). In spite of the fact that lipid substrates mostly support lipase production, in this case, the yeast produced a small amount of lipolytic enzymes. The fat substrate was most likely a complex carbon source for this yeast.

However, by adding Tween 80 to the medium, an increase in lipase activity to 0.73 nmol/min mL can be observed. Thus, for this strain, it can be assumed that in the presence of an emulsifier, the waste fat was used more efficiently. A similar trend of lipase activities can be observed in other studied strains. It can be concluded that Tween partially contributed to better emulsification of the fat medium, thus showing higher lipase activity in these strains.

The highest lipolytic activity observed on pure fat medium was recorded in *M. chrysoperlae* strain 1158 1.13 nmol/min mL and in the yeast *M. sinensis* 1244 with a value of 1.05 nmol/min mL. Moreover, the highest enzymatic activity of lipases was observed in this strain (*M. sinensis* 1244) on media containing Tween 80, where it was 1.30 nmol/min mL. From these experiments, a positive effect of the emulsifying properties of Tween 80 can be readily observed.

However, compared to other yeasts, the measured enzymatic activities of *Metschnikowia* yeast lipases were relatively small. In the yeast *Y. lipolytica*, after 72 h of cultivation, the production of extracellular lipases was determined to be 2.8 ∙ 10^4^ nmol/min ∙ ml, in its mutant strains, the production increased even up to 1 × 10^6^ nmol min mL [34]. We could observe that majority of yeasts show higher values of lipase activity during the later exponential phase.

A possible explanation of lower lipase activities could be the fact that the activity was not observed during the later exponential phase of the growth curve but at the end of the stationary phase of growth. This finding is also supported by the amounts of residual fat that were determined after the supernatant cultivation. We found that in some strains, fat was used up to 80%. However, on average, fat was used at 60%.

Previous studies have shown that olive oil is a possible carbon source substrate that can increase lipase synthesis. It contains approximately 55–83% oleic acid, which acts as an inhibitor for the production of a gene encoding lipase synthesis [33,34].

The addition of an emulsifier or the addition of oleic acid to the medium can induce an increased production of lipases in yeasts of the genus *Metschnikowia* and thus a better use of animal fat for the production of lipid metabolites. Higher amounts of lipases can break down TAG from animal fat, and the released glycerol will be used as a carbon source. From the results mentioned above, it can be seen that yeasts can utilize glycerol and have excellent production properties of both biomass and lipid metabolites. Therefore, the yeast conversion of crude processed animal fat into value-added products is a valuable process for the food industry.

### 3.5. Rapid Analysis of Lipids during Cultivation by Raman Spectroscopy

Fatty acid profiles were measured by GC. Simultaneously, we monitored the metabolic states of yeasts in order to optimize the cultivation process for biotechnological applications using Raman spectroscopy.

Raman spectroscopy offers a powerful alternative analytical method for the detection and identification of different substances in biological samples, such as bacteria, algae, and yeast. Raman spectroscopy can be exploited in instances where fast and accurate monitoring/determination of samples is required [35,36].

Here, attention is given to the oil-producing yeast strain genus *Metschnikowia* to exploit its potential applications in the biotechnology field. In order to utilize selected microorganisms for efficient biotechnological production, the influence of different cultivation parameters (such as the effects of temperature regime and medium composition) on cells can be monitored using different instrumentation [9].

In this study, Raman spectroscopy was used for monitoring changes in saturation degree of fatty acids (iodine number) in yeast cells. Raman spectroscopy can be utilized for fast and accurate lipids estimation as the intensity ratios of specific, selected Raman bands (lipid CH2 scissoring 1.445 cm^−1^, lipid C=C stretching 1.656 cm^−1^). Raman spectroscopy proved to be a very efficient tool for the rapid quantitative/qualitative analyses of oil produced by yeast. It can take several minutes [12,13].

The figure (Figure S1) shows the Raman spectra of the yeast *M. sinensis* 1244 cultivated on glycerol medium at two C/N ratios, 97 and 150. The work compared the results obtained from gas chromatography and Raman spectroscopy using iodine number (Table S4). It can be seen from the figure and the table that the yeast *M. sinensis* had a similar representation of saturated and unsaturated fatty acids on both mentioned media. This is also proven by the values of iodine numbers, which differ only minimally. We know from the results of gas chromatography that the yeast *M. sinensis* 1244 had a similar proportion of fatty acids and their amount in both culture media. In a previous study, Raman spectroscopy was successfully used to monitor unsaturation during cultivation on glucose media [9].

On the contrary is the case of *M. pulcherrima* 1232 and *M. sinensis* 1244 cultured on the same media with glycerol of C/N ratio 150. In (Figure S2) and (Table S5), it is seen that greater quantities of unsaturated fatty acids were produced by yeast *M pulcherrima* 1232 versus *M. sinensis* 1244. This is also evidenced by the iodine number calculated from the Raman spectrum. The work shows only illustrative examples due to the large number of spectra obtained.

By detecting the presence of fatty acids by the iodine number method [13], it was demonstrated that the results obtained from the Raman spectra approximately corresponded to the results obtained by gas chromatography with slight deviations. Thus, the results support the use of Raman spectroscopy in the analysis of microbial lipids already during the culture process, as this method provides a rapid and relatively accurate estimate of the unsaturation of the lipids produced. Thanks to Raman spectroscopy, we can quickly estimate the success or failure of targeted lipid production by manipulating culture conditions [36].

## 4. Conclusions

The aim of the study was primarily focused on the production and analysis of lipids under different conditions of nutritional and temperature stress [37]. The main focus of this study was to test the possible utilization of mixed waste animal fat by *Metschnikowia* yeasts for the production of higher-value lipids. Because animal fat—after treatment—provides yeast with glycerol as a rapid source of energy, specialized experiments were performed to verify the use of glycerol by selected yeasts of the genus *Metschnikowia*. The use of glycerol further corresponds to the possibility of utilizing waste glycerol produced in the production of biofuels.

All strains showed the ability to assimilate the substrates used as a carbon source and formed the desired metabolites. Yeasts were cultivated under reduced temperature conditions (15 °C) for 14 days to ensure the most suitable conditions for controlled lipid overproduction. Sub-optimum temperatures and higher carbon-to-nitrogen ratios have been shown to be most advantageous for achieving maximum lipid yields. The highest lipid production was observed on glycerol media, where the content of accumulated lipids in *M. pulcherrima* 1232 cells was 36.3% (C/N 150).

By using waste substrates, the yeasts of the genus *Metschnikowia* produced such lipids, which were rich mainly in C16 and C18 fatty acids. That indicates their use in the production of biofuels [38]. Glycerol medium induced to a large extent the production of unsaturated fatty acids with a high proportion of linoleic acid. Glycerol assimilation also led to the production of significant additional polyunsaturated FA to a significantly higher extent compared to glucose and waste animal fat. Waste crude fat is a good source of glycerol and free fatty acids after hydrolysis.

By analyzing the profile of fatty acids produced by the yeasts of the genus *Metschnikowia*, a certain similarity was observed in all strains in the representation and approximate % of the fatty acid content. A notable exception was the strain *M. sinensis* 1244, which showed some difference on all culture media, regardless of the carbon source used. Among all strains, it was the highest producer of α-linolenic acid on each medium, which belongs to the important Ω-3 fatty acids, and at the same time, it was almost always the lowest producer of oleic acid.

The achieved lipid yields therefore increase the oleogenic potential of *Metschnikowia* yeast in the biotechnology industry, as the ability to produce high lipids by using a waste substrate—glycerol or crude fat—has been demonstrated, which appears to be very economically and environmentally beneficial, as it would reduce waste in the environment. Moreover, by modifying crude animal fat or with higher production of lipase enzymes, yeasts should in turn utilize fat more efficiently. Our results show that yeast not only utilizes animal fat but also converts it into lipid products with a different fatty acid profile than the original composition.

Moreover, the use of cultivation in a reduced-temperature environment is also of great importance, as this would significantly reduce the risk of contamination.

## Figures and Tables

**Table 1:** Composition of carbon and nitrogen source at different C/N ratios.

C/N Ratio	Carbon Source (g/L)	Bacteriological Pepton (g/L)	Yeast Extract (g/L)
24	Glucose 30	1.7	3.4
97	Glucose 30	0.68	0.34
150	Glucose 30	0.34	0.34
24	Glycerol 90	5.0	10.0
97	Glycerol 90	2.0	1.0
150	Glycerol 90	1.0	1.0

**Table 2:** Carbon and nitrogen sources from various materials at different C/N ratios.

C/N Ratio	Crude fat as C Source (g/L)	Bacteriological Pepton (g/L)	Yeast Extract (g/L)
24	47	10.0	5.0
97	45	1.0	2.0
150	50	1.0	1.0

**Table 3:** Lipids and biomass production at laboratory (25 °C) and cold temperature (15 °C).

Strain	Biomass (g/L) /15 °C	Lipid Content (%) ^a^ /15 °C	Biomass (g/L) /25 °C	Lipid Content (%) ^a^ /25 °C
*M. pulcherrima* 145	4.3 ± 0.2	4.1 ± 0.2	5.1 ± 0.3	3.4 ± 0.4
*M. pulcherrima* 147	6.2 ± 0.5	3.8 ± 0.3	8.9 ± 0.7	3.3 ± 0.3
*M. pulcherrima* 149	8.3 ± 0.6	4.7 ± 0.4	11.2 ± 0.7	4.5 ± 0.4
*M. andauensis* 129	5.4 ± 0.3	4.0 ± 0.4	5.7 ± 0.3	3.1 ± 0.3
*M. chrysoperlae* 1158	10.9 ± 0.8	7.0 ± 1.4	11.3 ± 1.5	6.8 ± 1.3
*M. pulcherrima* 1232	7.7 ± 0.6	7.8 ± 0.9	11.1 ± 1.2	6.3 ± 0.8
*M. fructicola* 1235	7.9 ± 0.6	6.5 ± 0.4	9.4 ± 0.7	5.6 ± 0.7
*M. andauensis* 1241	8.6 ± 0.9	7.9 ± 1.6	13.4 ± 1.1	7.1 ± 0.6
*M. sinensis* 1244	9.1 ± 1.3	7.8 ± 0.9	10.1 ± 0.6	5.8 ± 1.7
*M. zizyphicola* 1247	7.5 ± 1.1	6.0 ± 0.9	10.9 ± 1.5	5.1 ± 0.6
*M. shanxiensis* 1250	8.3 ± 0.6	6.5 ± 1.2	9.7 ± 0.3	5.6 ± 1.3

^a^—Lipid (g/L)/Biomass (g/L) dry weight × 100.

**Table 4:** Selected fatty acid profile (%) of lipid content produced by *Metschnikowia andauensis 1241* cultivated on optimal glucose medium.

Fatty Acid	Laboratory Temperature	Cold Temperature
C15:0	0.56	0.35
C16:0	6.66	8.49
C16:1n7	5.46	5.45
C18:0	0.94	1.03
C18:1	65.10	50.96
C18:2	17.63	28.31
C18:3n6	-	0.10
C18:3n3	0.77	1.92
SFA	8.16	11.2
MUFA	71.39	56.41
PUFA	20.45	32.39

**Table 5:** Biomass and lipids production at low temperature cultivated on glucose medium with different C/N ratio.

Strain		C/N 24	C/N 97	C/N 150
*M. pulcherrima* 145	Biomass (g/L)	4.5 ± 0.3	4.0 ± 0.2	3.8 ± 0.3
	Lipid content (%) ^a^	4.6 ± 0.2	5.2 ± 0.4	6.5 ± 0.6
*M. pulcherrima* 147	Biomass (g/L)	6.6 ± 0.5	6.8 ± 0.5	6.4 ± 0.7
	Lipid content (%) ^a^	4.1 ± 0.4	4.3 ± 0.5	4.8 ± 0.4
*M. pulcherrima* 149	Biomass (g/L)	8.8 ± 1.0	8.0 ± 0.9	8.0 ± 1.0
	Lipid content (%) ^a^	5.2 ± 0.3	6.0 ± 0.6	7.2 ± 0.5
*M. andauensis* 129	Biomass (g/L)	5.5 ± 0.4	5.1 ± 0.4	5.0 ± 0.6
	Lipid content (%) ^a^	4.4 ± 0.5	4.8 ± 0.4	5.8 ± 0.7
*M. chrysoperlae* 1158	Biomass (g/L)	9.3 ± 0.8	6.85 ± 0.7	7.1 ± 0.7
	Lipid content (%) ^a^	6.6 ± 0.9	12.33 ± 1.5	13.6 ± 0.9
*M. pulcherrima* 1232	Biomass (g/L)	8.3 ± 0.7	7.0 ± 0.5	6.5 ± 0.7
	Lipid content (%) ^a^	8.2 ± 1.1	14.6 ± 1.3	15.4 ± 1.0
*M. fructicola* 1235	Biomass (g/L)	11.6 ± 1.3	11.2 ± 1.5	7.6 ± 1.2
	Lipid content (%) ^a^	6.2 ± 0.5	6.7 ± 1.2	6.5 ± 0.7
*M. andauensis* 1241	Biomass (g/L)	9.6 ± 0.8	9.2 ± 1.3	8.2 ± 1.1
	Lipid content (%) ^a^	7.5 ± 0.4	16.9. ± 1.5	19.6 ± 1.6
*M. sinensis* 1244	Biomass (g/L)	10.8 ± 1.2	10.3 ± 1.6	9.9 ± 1.0
	Lipid content (%) ^a^	8.8 ± 0.9	14.4 ± 1.5	16.9 ± 1.5
*M. zizyphicola* 1247	Biomass (g/L)	9.5 ± 1.1	7.1 ± 0.6	7.0 ± 0.6
	Lipid content (%) ^a^	7.1 ± 0.9	14.0 ± 1.5	16.0 ± 1.2
*M. shanxiensis* 1250	Biomass (g/L)	8.9 ± 0.8	7.7 ± 0.6	7.3 ± 0.6
	Lipid content (%) ^a^	7.3 ± 0.9	16.8 ± 1.3	16.1 ± 1.2

^a^—Lipid (g/L)/Biomass (g/L) dry weight × 100.

**Table 6:** Production biomass and lipids at low temperature cultivated on glycerol medium with different C/N ratio.

Strain		C/N 24	C/N 97	C/N 150
*M. pulcherrima* 145	Biomass (g/L)	11.5 ± 0.8	6.0 ± 0.5	5.1 ± 0.4
	Lipid content (%) ^a^	3.8 ± 0.5	3.8 ± 0.4	4.5 ± 0.5
*M. pulcherrima* 147	Biomass (g/L)	11.4 ± 1.0	7.5 ± 0.6	7.2 ± 0.8
	Lipid content (%) ^a^	3.2 ± 0.3	3.4 ± 0.5	6.5 ± 0.7
*M. pulcherrima* 149	Biomass (g/L)	13.2 ± 1.2	8.9 ± 1.0	9.2 ± 0.9
	Lipid content (%) ^a^	5.0 ± 0.6	6.1 ± 0.5	11.3 ± 0.8
*M. andauensis* 129	Biomass (g/L)	11.8 ± 0.8	6.6 ± 0.8	6.1 ± 0.6
	Lipid content (%) ^a^	2.1 ± 0.3	4.0 ± 0.3	4.2 ± 0.5
*M. chrysoperlae* 1158	Biomass (g/L)	25.3 ± 2.1	19.9 ± 2.1	17.3 ± 1.9
	Lipid content (%) ^a^	11.0 ± 1.2	13.9 ± 1.5	18.1 ± 2.0
*M. pulcherrima* 1232	Biomass (g/L)	19.4 ± 1.8	19.7 ± 1.9	16.3 ± 1.2
	Lipid content (%) ^a^	17.8 ± 1.3	17.9 ± 1.5	36.3 ± 2.8
*M. fructicola* 1235	Biomass (g/L)	15.5 ± 1.4	11.5 ± 1.0	11.4 ± 1.2
	Lipid content (%) ^a^	6.7 ± 0.7	10.0 ± 0.7	15.3 ± 1.7
*M. andauensis* 1241	Biomass (g/L)	26.1 ± 1.9	18.6 ± 1.5	17.9 ± 1.5
	Lipid content (%) ^a^	13.0 ± 1.4	17.7 ± 2.0	17.0 ± 1.6
*M. sinensis* 1244	Biomass (g/L)	27.7 ± 2.5	24.3 ± 2.2	22.4 ± 2.0
	Lipid content (%) ^a^	16.6 ± 1.8	27.5 ± 2.3	29.9 ± 2.7
*M. zizyphicola* 1247	Biomass (g/L)	16.9 ± 1.7	21.1 ± 1.8	26.8 ± 2.8
	Lipid content (%) ^a^	35.5 ± 4.0	21.3 ± 2.5	18.4 ± 2.0
*M. shanxiensis* 1250	Biomass (g/L)	21.5 ± 2.2	15.5 ± 1.2	14.8 ± 1.5
	Lipid content (%) ^a^	12.2 ± 1.3	12.9 ± 1.7	18.3 ± 1.9

^a^—Lipid (g/L)/Biomass (g/L) dry weight × 100.

**Table 7:** Production biomass and lipids at low temperature cultivated on crude animal fat medium with different C/N ratio.

Strain		C/N 24	C/N 97	C/N 150
*M. pulcherrima* 145	Biomass (g/L)	5.8 ± 0.7	3.8 ± 0.4	3.8 ± 0.3
	Lipid content (%) ^a^	8.2 ± 0.6	10.6 ± 0.8	11.6 ± 0.9
*M. pulcherrima* 147	Biomass (g/L)	8.4 ± 0.8	2.1 ± 0.3	2.0 ± 0.2
	Lipid content (%) ^a^	6.5 ± 0.5	7.5 ± 0.5	11.1 ± 0.9
*M. pulcherrima* 149	Biomass (g/L)	5.8 ± 0.7	3.3 ± 0.1	2.7 ± 0.3
	Lipid content (%) ^a^	9.0 ± 0.7	8.3 ± 1.0	9.1 ± 0.8
*M. andauensis* 129	Biomass (g/L)	5.6 ± 0.8	3.4 ± 0.4	2.9 ± 0.3
	Lipid content (%) ^a^	8.8 ± 0.7	9.7 ± 0.7	10.6 ± 0.9
*M. chrysoperlae* 1158	Biomass (g/L)	14.5 ± 1.8	12.0 ± 1.1	11.8 ± 1.5
	Lipid content (%) ^a^	7.1 ± 0.6	9.7 ± 1.0	10.7 ± 0.9
*M. pulcherrima* 1232	Biomass (g/L)	7.7 ± 0.9	3.7 ± 0.5	3.5 ± 0.5
	Lipid content (%) ^a^	6.7 ± 0.5	8.9 ± 1.0	12.5 ± 1.5
*M. fructicola* 1235	Biomass (g/L)	5.7 ± 0.4	3.0 ± 0.4	2.6 ± 0.4
	Lipid content (%) ^a^	6.5 ± 0.8	7.4 ± 0.7	12.6 ± 1.5
*M. andauensis* 1241	Biomass (g/L)	15.7 ± 1.8	8.5 ± 0.7	8.4 ± 0.6
	Lipid content (%) ^a^	13.9 ± 1.2	14.4 ± 1.3	14.0 ± 1.5
*M. sinensis* 1244	Biomass (g/L)	8.8 ± 1.0	7.7 ± 0.9	4.1 ± 0.6
	Lipid content (%) ^a^	6.7 ± 0.8	7.7 ± 0.7	8.8 ± 0.9
*M. zizyphicola* 1247	Biomass (g/L)	7.2 ± 0.6	5.3 ± 0.4	4.3 ± 0.3
	Lipid content (%) ^a^	11.5 ± 1.3	12.9 ± 1.5	20.4 ± 2.0
*M. shanxiensis* 1250	Biomass (g/L)	5.5 ± 0.6	3.8 ± 0.5	3.4 ± 0.5
	Lipid content (%) ^a^	9.5 ± 1.1	12.4 ± 1.4	10.2 ± 1.1

^a^—Lipid (g/L)/Biomass (g/L) dry weight × 100.

**Table 8:** Enzymatic activity of lipase (nmol/mL.min) on crude animal fat medium (47 g/L) and medium with addition of Tween 80 medium with added Tween 80 emulsifier and amount of residual fat (from supernatant) after 14 days of cultivation on low temperature.

Strain	Without Tween 80			With Tween 80		
	Biomass (g/L)	Lipase Activity (nmol/mL·min)	Residual Animal Fat (g/L)	Biomass (g/L)	Lipase Activity (nmol/mL·min)	Residual Animal fat (g(L))
*M. pulcherrima* 145	6.1 ± 0.7	0.79 ± 0.00	22.56 ± 1.8	6.4 ± 0.6	0.83 ± 0.00	20.43 ± 1.9
*M. pulcherrima* 147	8.8 ± 0.7	0.81 ± 0.01	17.39 ± 1.5	9.0 ± 0.7	0.85 ± 0.00	16.06 ± 1.3
*M. pulcherrima* 149	6.3 ± 0.8	1.02 ± 0.01	14.10 ± 1.6	7.0 ± 0.5	1.03 ± 0.00	13.54 ± 1.4
*M. andauensis* 129	5.9 ± 0.7	0.99 ± 0.00	26.92 ± 2.2	6.5 ± 0.6	1.01 ± 0.00	24.67 ± 2.0
*M. chrysoperlae* 1158	15.3 ± 1.4	1.13 ± 0.01	10.81 ± 0.7	16.2 ± 1.2	1.13 ± 0.01	8.87 ± 0.6
*M. pulcherrima* 1232	8.1 ± 0.8	0.78 ± 0.01	19.74 ± 2.2	8.6 ± 0.6	1.00 ± 0.01	17.13 ± 1.5
*M. fructicola* 1235	6.0 ± 0.5	0.80 ± 0.00	16.92 ± 0.9	7.2 ± 0.5	0.94 ± 0.01	15.94 ± 1.0
*M. andauensis* 1241	15.9 ± 1.5	0.88 ± 0.00	13.16 ± 0.8	16.3 ± 0.9	0.91 ± 0.00	13.10 ± 1.0
*M. sinensis* 1244	9.3 ± 1.0	1.05 ± 0.01	12.69 ± 0.5	10.7 ± 0.9	1.30 ± 0.02	9.08 ± 0.6
*M. zizyphicola* 1247	7.8 ± 0.7	0.57 ± 0.00	18.8 ± 1.1	8.6 ± 1.0	0.73 ± 0.00	15.30 ± 1.0
*M. shanxiensis* 1250	6.2 ± 0.5	0.62 ± 0.00	20.21 ± 1.3	7.4 ± 0.7	0.79 ± 0.00	17.14 ± 1.1

## Supplementary Materials

The following are available online at https://www.mdpi.com/article/10.3390/microorganisms9112295/s1, Figure S1: Raman scattering spectrum of intracellular lipids of the yeast *M. sinensis* MS 1244 on two different media. Figure S2. Raman scattering spectrum of intracellular lipids of the yeast *M. pulcherrima 1232 and M. sinensis* MS 1244 on the same media. Table S1. Composition of fatty acids (%) of glucose medium and C/N ratio 24, 97 and 150. Table S2. Composition of fatty acids (%) of glycerol medium and C/N ratio 24, 97 and 150. Table S3. Composition of fatty acids (%) of crude animal fat and its conversion in yeast fatty acids. Yeasts were cultivated in medium with crude fat and C/N ratio 24, 97 and 150. Table S4. Representation of SFA, MUFA and PUFA (%) in the yeast *M. sinensis 1244* cultivated on glycerol media with C/N ratio 97 and 150 and calculated iodine number from the Raman spectrum (according to equation at [13]). Table S5. Representation of SFA, MUFA and PUFA (%) in the yeast M. pucherrima 1232 and M. sinensis 1244 cultivated on glycerol media with a C/N ratio of 150 and the calculated iodine value from the Raman spectrum (according to equation at [13]).
