# Supplementary material for: Use of Waste Substrates for the Lipid Production by Yeasts of the Genus Metschnikowia—Screening Study
Source: Microorganisms. 2021 Nov 4;9(11):2295. doi: 10.3390/microorganisms9112295 (PMC8620705; doi:10.3390/microorganisms9112295)
Supplement: Supplementary file 1 [file microorganisms-09-02295-s001.zip › microorganisms-1427707-supplementary.pdf]

microorganisms

Suplementary material

Use Of Waste Substrates For The Lipid Production By Yeasts Of The Genus *Metschnikowia*- screening study

Andrea Němcová [1*], Martin Szotkowski [1], Ota Samek [2], Linda Cagáňová [1], Matthias Sipiczki [3] and Ivana Márová [1]

[1] Faculty of Chemistry, Brno University of Technology, Purkyňova 464/118, 612 00 Brno, Czech Republic
[2] Institute of Scientific Instruments of the Czech Academy of Sciences, Královopolská 147, 612 64 Brno, Czech Republic
[3] Department of Genetics and Applied Microbiology, Faculty of Science and Technology, University of Debrecen, Egyetem tér 1,4032 Debrecen, Hungary

* Correspondence: andrea.nemcova@fch.vut.cz; Tel.: +420-541-149-419

Contents:

**Figure S1**	Raman scattering spectrum of intracellular lipids of the yeast *M. sinensis* MS 1244 on two different media.
**Figure S2**	Raman scattering spectrum of intracellular lipids of the yeast *M. pulcherrima 1232 and M. sinensis* MS 1244 on the same media
**Table S1**	GC analysis - composition of fatty acids (%) of glucose medium and C/N ratio 24, 97 and 150.
**Table S2**	GC analysis - Composition of fatty acids (%) of glycerol medium and C/N ratio 24, 97 and 150.
**Table S3**	GC analysis - Composition of fatty acids (%) of crude animal fat and its conversion in yeast fatty acids. Yeasts were cultivated in medium with crude fat and C/N ratio 24, 97 and 150.
**Table S4**	Representation of SFA, MUFA and PUFA (%) in the yeast *M. sinensis 1244* cultivated on glycerol media with C/N ratio 97 and 150 and calculated iodine number from the Raman spectrum (according to equation at [13])
**Table S5**	Representation of SFA, MUFA and PUFA (%) in the yeast *M. pucherrima 1232* and *M. sinensis 1244* cultivated on glycerol media with a C/N ratio of 150 and the calculated iodine value from the Raman spectrum (according to equation at [13])

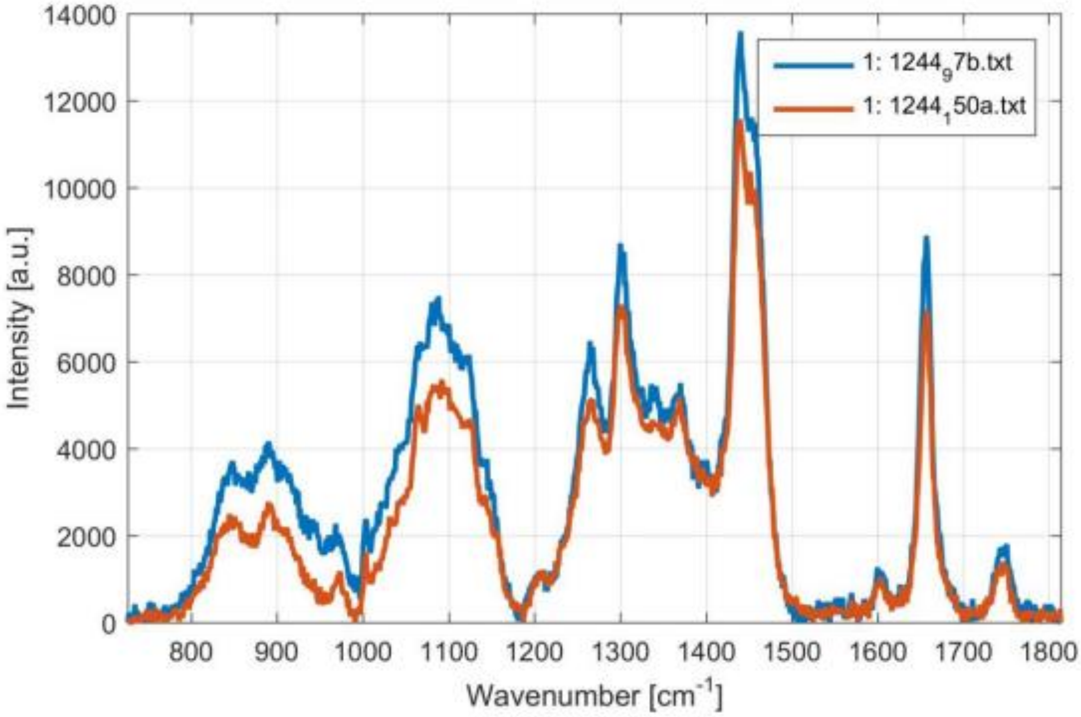

Figure S1. Raman scattering spectrum of intracellular lipids of the yeast *M. sinensis* MS 1244 on two different media. Legend: *Metschnikowia sinensis* 1244 cultivated on medium with C/N ratio 97 (1244_97, blue trace) and on medium with C/N ratio 150 (1244_150, red trace).

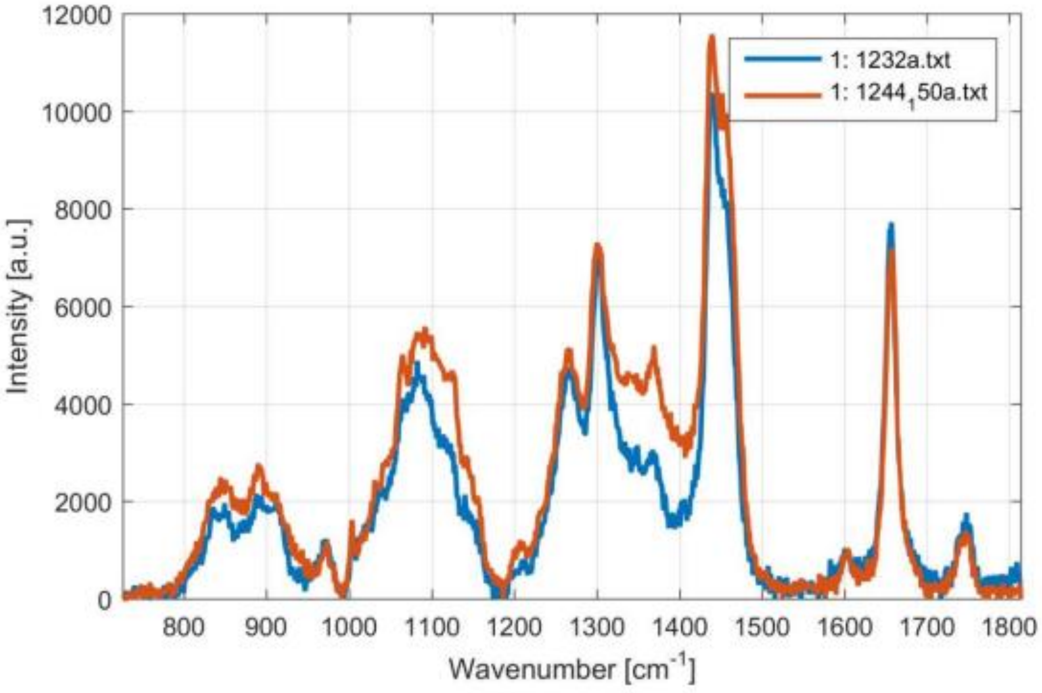

Figure S2: Raman scattering spectrum of intracellular lipids of the yeast *M. pulcherrima 1232 and M. sinensis* MS 1244 on the same media. Legend: *Metschnikowia pulcherrima* 1232 cultivated on medium with C/N ratio 150 (1232_150, blue trace) and *M. sinensis* 1244 cultivated on medium with C/N ratio 150 (1244_150, red trace).

Table S1: Composition of fatty acids (%) of glucose medium and C/N ratio 24, 97 and 150.

C/N 24 glucose											
Fatty acid (%)	145	147	149	129	1158	1232	1235	1241	1244	1247	1250
C16:0	7.1	10.3	9.1	8.8	8.9	8.7	9.3	9.6	19.6	9.3	8.8
C16:1	9.3	8.1	7.9	9.5	8.4	6.5	5.9	4.3	1.8	5.5	8.3
C18:0	4.7	6.7	6.4	4.5	3.5	4.3	5.1	4.7	15.1	3.1	4.8
C18:1	51.8	61.6	57.3	47.3	66.7	65.2	52.8	70.1	48.3	67.2	63.2
C18:2	26.3	12.8	18.8	27.6	12.3	14.7	25.9	9.8	14.7	14.5	13.6
C18:3	0.0	0.0	0.1	0.0	0.0	0.0	0.1	0.7	0.0	0.0	0.1
others	0.8	0.5	0.4	2.3	0.2	0.6	0.9	0.8	0.5	0.4	1.2
C/N 97 glucose											
Fatty acid (%)	145	147	149	129	1158	1232	1235	1241	1244	1247	1250
C16:0	6.1	9.2	7.5	7.7	8.0	7.9	8.1	7.8	16.5	8.8	7.6
C16:1	9.7	8.3	8.3	9.6	8.6	7.4	6.5	4.5	2.5	5.9	8.7
C18:0	3.6	5.9	5.0	2.9	1.9	3.1	3.5	3.0	13.1	2.0	3.1
C18:1	53.2	63.3	59.1	49.7	68.4	66.1	53.7	73.7	51.3	68.1	65.4
C18:2	27.2	13.1	19.7	29.3	13	15.2	27.9	10.6	16.3	15.1	14.7
C18:3	0.1	0.0	0.1	0.0	0.0	0.0	0.1	0.2	0.0	0.0	0.1
others	0.1	0.2	0.3	0.8	0.1	0.3	0.2	0.2	0.3	0.1	0.4
C/N 150 glucose											
Fatty acid (%)	145	147	149	129	1158	1232	1235	1241	1244	1247	1250
C16:0	6.7	9.6	6.3	7.9	9.4	8.3	8.6	7.7	18.3	9.2	8.0
C16:1	10.3	8.4	8.6	10.8	8.9	6.5	6.2	3.3	3.1	6.2	8.6
C18:0	4.1	6.5	4.2	3.1	1.5	3.2	3.7	2.8	14.6	3.3	3.8
C18:1	51.9	61.8	62.4	47.2	67.3	68.1	57.2	74.5	49.8	66.2	65
C18:2	26.8	13.3	18.4	26	12.8	13.7	23.4	11.2	13.6	14.7	13.9
C18:3	0.0	0.0	0.0	0.1	0.0	0.0	0.1	0.4	0.0	0.0	0.1
others	0.2	0.4	0.1	4.9	0.1	0.2	0.8	0.1	0.6	0.4	0.6

Legends: 1158 – Metschnikowia chrysoperlae, 1232 – Metschnikowia pulcherrima, 1235 - Metschnikowia fructicola, 1241 - Metschnikowia andauensis, 1244 – Metschnikowia sinensis, 1247 – Metschnikowia zizyphicola, 1250 – Metschnikowia shanxiensis, 145 – Metschnikowia pulcherrima, 147 – Metschnikowia pulcherrima, 149 – Metschnikowia pucherrima, 129 – Metschnikowia andauensis

Table S2: Composition of fatty acids (%) of glycerol medium and C/N ratio 24, 97 and 150.

C/N 24 glycerol											
Fatty acid (%)	145	147	149	129	1158	1232	1235	1241	1244	1247	1250
C16:0	5.6	9.8	6	7.5	7.8	7.6	6.9	8.3	14.3	11.4	6.7
C16:1	8.3	6.2	7.7	7.1	8.6	7.4	5.8	4.1	2.2	6.8	8.4
C18:0	4.3	5.8	3.5	4.0	1.8	2.3	3.1	3.4	14	1.7	2.6
C18:1	46.6	57.1	50.1	31.7	66.4	58.4	43.9	67.7	42.9	69.5	60.6
C18:2	31.9	18.2	29.6	44.3	13.8	21.6	36.8	13.9	21.6	8.4	17.5
C18:3	1.1	1.5	1.6	3.1	0.9	1.9	2.4	1.8	4.4	1.6	3.4
C20:4	0.2	0.1	0.8	1.4	0.2	0.1	0.2	0.1	0.0	0.1	0.2
others	2	1.3	0.7	0.9	0.5	0.7	0.9	0.7	0.6	0.5	0.6
C/N 97 glycerol											
Fatty acid (%)	145	147	149	129	1158	1232	1235	1241	1244	1247	1250
C16:0	5.9	9.8	6.6	7.7	7.8	8.6	7.5	8.4	14.5	8.7	6.9
C16:1	8.1	6.0	7.7	6.8	7.1	7.2	6.2	4.2	2.4	5.6	8.5
C18:0	5.5	6.1	4.5	3.8	2.4	3.8	3.6	3.5	13.6	2.1	3.0
C18:1	45.5	59.7	54.1	28.9	66.3	60.9	48.6	70.5	44.1	65.6	62.3
C18:2	33.4	17.2	25.7	46.2	13.6	17.6	33.2	12.3	22.9	17.6	16
C18:3	1.2	0.9	0.8	2.4	1.2	1.5	0.6	0.7	1.8	0.2	2.4
C20:4	0.1	0.1	0.2	0.8	0.2	0.0	0.1	0.1	0.0	0.0	0.1
others	0.3	0.2	0.4	3.4	1.4	0.4	0.2	0.3	0.7	0.2	0.8
C/N 150 glycerol											
Fatty acid (%)	145	147	149	129	1158	1232	1235	1241	1244	1247	1250
C16:0	6.4	10.0	7.4	7.7	7.7	9.3	8.2	8.8	14.6	7.3	6.8
C16:1	7.9	6.3	7.8	4.9	6.5	7.3	6.7	3.9	2.2	3.8	8.1
C18:0	5.8	6.2	4.9	3.7	3.7	5.1	3.7	3.9	14.0	1.9	3.4
C18:1	44.9	58.8	53.6	26.8	66.8	60.1	47.2	69.2	43.2	65.5	61.1
C18:2	33.1	16.9	24.2	46.9	13.6	16.1	31.6	11.8	23.4	18.7	16.5
C18:3	1.3	1.3	1.2	0.2	1.0	0.9	1.7	1.5	1.7	2.3	3.7
C20:4	0.2	0.1	0.2	1.3	0.3	0.0	0.1	0.2	0.0	0.1	0.1
others	0.4	0.4	0.7	8.5	0.4	1.2	0.8	0.7	0.9	0.4	0.3

Legends: 1158 – Metschnikowia chrysoperlae, 1232 – Metschnikowia pulcherrima, 1235 - Metschnikowia fructicola, 1241 - Metschnikowia andauensis, 1244 – Metschnikowia sinensis, 1247 – Metschnikowia zizyphicola, 1250 – Metschnikowia shanxiensis, 145 – Metschnikowia pulcherrima, 147 – Metschnikowia pulcherrima, 149 – Metschnikowia pucherrima, 129 – Metschnikowia andauensis

Table S3: Composition of fatty acids (%) of crude animal fat and its conversion in yeast fatty acids. Yeasts were cultivated in medium with crude fat and C/N ratio 24, 97 and 150.

C/N 24											
Fatty acid (%)	Animal fat	1158	1232	1235	1241	1244	1247	1250	145	147	149	129
C16:0	23.3	6.4	13.3	7.7	9.0	10.4	6.6	7.3	6.5	8.9	7.1	9.1
C16:1	6.0	3.2	4.0	4.8	4.7	2.8	4.3	4.9	4.3	4.5	5.6	4.5
C18:0	18.4	1.4	5.0	1.7	2.5	4.0	1.3	1.6	1.0	1.7	1.4	2.2
C18:1	39.1	74.8	57.1	70.1	68.8	57.5	74.8	69.8	72.9	69.6	65.8	68.6
C18:2	6.7	10.7	18.5	11.9	11.2	18.7	10.1	13.7	12.4	11.9	16.1	12.3
C18:3	0.3	0.9	0.9	1.0	1.1	4.7	0.9	1.2	1.1	0.8	1.4	0.9
Others	6.0	2.6	1.2	2.8	2.7	1.9	2.0	1.5	1.8	2.6	2.6	2.4
C/N 97											
	Animal fat	1158	1232	1235	1241	1244	1247	1250	145	147	149	129
C16:0	23.3	9.5	13.1	10.3	9.0	12.4	6.8	6.8	7.1	11.4	9.6	10.9
C16:1	6.0	3.7	4.1	4.0	6.2	2.3	4.9	4.5	3.7	4.8	1.9	5.2
C18:0	18.4	3.0	4.0	2.2	3.6	5.4	1.1	1.1	1.5	2.0	1.8	2.7
C18:1	39.1	71.5	56.0	63.3	70.2	51.0	73.6	71.5	72.3	61.4	67.4	63.2
C18:2	6.7	9.2	21.8	16.2	10.0	21.2	10.6	13.4	13.4	16.5	17.0	14.3
C18:3	0.3	0.7	0.6	0.1	0.5	5.9	1.1	1.4	1.1	1.5	1.3	1.2
Others	6.0	2.4	0.4	3.9	0.5	1.8	1.9	1.3	0.9	2.4	1.0	2.5
C/N 150											
	Animal fat	1158	1232	1235	1241	1244	1247	1250	145	147	149	129
C16:0	23.3	9.3	12.6	10.1	8.0	12.3	4.4	8.0	8.5	13.1	8.9	11.9
C16:1	6.0	3.7	3.9	4.0	7.1	2.4	3.1	4.8	4.8	5.9	4.0	5.3
C18:0	18.4	2.7	4.3	2.0	1.3	5.3	4.1	1.6	1.6	1.9	2.3	3.6
C18:1	39.1	70.2	50.5	58.7	71.3	50.2	65.8	67.9	71.4	58.1	63.0	58.1
C18:2	6.7	10.3	25.8	21.1	10.5	22.4	11.8	14.2	11.0	16.9	17.6	16.6
C18:3	0.3	0.8	1.4	1.4	1.3	5.7	3.7	1.6	1.1	1.9	1.6	1.7
Others	6.0	3.0	1.5	2.7	0.5	1.7	2.1	1.9	1.6	2.2	2.6	2.8

Legends: 1158 – Metschnikowia chrysoperlae, 1232 – Metschnikowia pulcherrima, 1235 - Metschnikowia fructicola, 1241 - Metschnikowia andauensis, 1244 – Metschnikowia sinensis, 1247 – Metschnikowia zizyphicola, 1250 – Metschnikowia shanxiensis, 145 – Metschnikowia pulcherrima, 147 – Metschnikowia pulcherrima, 149 – Metschnikowia pucherrima, 129 – Metschnikowia andauensis

Table S4: Representation of SFA, MUFA and PUFA (%) in the yeast *M. sinensis 1244* cultivated on glycerol media with C/N ratio 97 and 150 and calculated iodine number from the Raman spectrum (according to equation at [13])

Strain	medium	SFA (%)	MUFA (%)	PUFA (%)	Iodine number
1244	Gly C/N 97	18.38	54.54	27.08	80.77
1244	Gly C/N 150	18.12	53.69	28.19	81.57

Table S5. Representation of SFA, MUFA and PUFA (%) in the yeast *M. pucherrima 1232* and *M. sinensis 1244* cultivated on glycerol media with a C/N ratio of 150 and the calculated iodine value from the Raman spectrum (according to equation at [13])

Strain	medium	SFA (%)	MUFA (%)	PUFA (%)	Iodine number
1244	Gly C/N 150	18.12	53.69	28.19	75.74
1232	Gly C/N 150	14.63	67.99	17.39	97.15
